# Mediating mechanisms of the relationship between exposure to deprivation and threat during childhood and adolescent psychopathology: evidence from the Millennium Cohort Study

**DOI:** 10.1007/s00787-023-02289-3

**Published:** 2023-09-07

**Authors:** Ke Ning, Dawid Gondek, Snehal M. Pinto Pereira, Rebecca E. Lacey

**Affiliations:** 1 School of Public Health, LKS Faculty of Medicine, The University of Hong Kong, Hong Kong Special Administrative Region, China; 2 Research Department of Epidemiology and Public Health, University College London, London, England; 3 Swiss Centre of Expertise in Life Course Research (LIVES), University of Lausanne, Lausanne, Switzerland; 4 Division of Surgery and Interventional Science, University College London, London, England; 5 Population Health Research Institute, St George’s, University of London, London, England

**Keywords:** Deprivation, Threat, Psychopathology, Millennium Cohort Study, Cognitive ability, Emotion regulation

## Abstract

The key aim of our study was to examine pathways from exposure to childhood adversities (i.e., deprivation and threat) to adolescent psychopathology. The assessed mediating mechanisms included cognitive ability and emotion regulation, as proposed by the Dimensional Model of Adversity and Psychopathology (DMAP). The study comprised participants from the nationally representative Millennium Cohort Study. Latent scores for deprivation and threat were derived using confirmatory factor analysis from indicators collected when participants were at age of 9 months, 3 and 5 years. Cognitive ability was measured using the Verbal Similarities subscale of the British Ability Scales II at age 11, and emotion regulation was measured using emotion dysregulation subscale of the Child Social Behavioural Questionnaire at age 7. Psychopathology, defined as psychological distress, was assessed using the Kessler 6 scale at age 17. We conducted causal mediation analysis adjusting for multiple confounding factors. We did not find total effect of either exposure to deprivation or threat on psychological distress, but we did find significant indirect effects of exposure to deprivation on psychological distress via cognitive ability (− 0.11, 95% CI − 0.20 to − 0.05) and emotion regulation (0.03, 0.02 to 0.12), and exposure to threat on psychological distress via cognitive ability (− 0.04, − 0.07 to − 0.01) and emotion regulation (0.09, 0.03 to 0.15). The lack of associations between deprivation or threat and psychological distress may be due to reporting bias or developmental period of psychopathology. Results of mediation analysis partially support the DMAP but indicate limited benefits to reduce adolescent psychological distress by targeting cognitive ability or emotion regulation to those exposed to childhood adversities.

**Supplementary Information:**

The online version contains supplementary material available at 10.1007/s00787-023-02289-3.

## Introduction

Adverse childhood experiences, such as child maltreatment and parental mental health problems, exhibit strong, graded associations with a range of mental health outcomes throughout the life course [1–6]. The Dimensional Model of Adversity and Psychopathology (DMAP) distinguishes two underlying adversity dimensions—deprivation and threat [7, 8]. Deprivation is broadly defined as insufficient environmental complexity for a given developmental stage, with the primary emphasis on the lack of cognitive and social–relational stimulation, typically associated with neglect [7]. Children raised in families with low socioeconomic resources tend to be less exposed to language and cognitive stimulation at home [9–11]. Hence, researchers often rely on socioeconomic variables (e.g., maternal education, household income) as indicators of deprivation. High-quality information about home environment is rarely available in population-based observational studies [12, 13]. We have taken a similar approach in our study, defining deprivation in the context of socioeconomic resources, serving as a proxy for deficits in social and cognitive inputs at home. It has been argued that separate dimensions of deprivation may have unique developmental consequences and pathways to psychopathology, which are important to understand to inform potential interventions [14].

Threat involves experiences of threat or harm to the child, such as exposure to abuse or violence [7]. The central tenet of the DMAP model is that deprivation and threat often co-occur; however, DMAP posits that at least partially distinct developmental pathways link these dimensions of adversity to psychopathology [7]. In this way, the model proposes testable hypotheses, which can subsequently help to identify mechanisms serving as potential targets for secondary interventions. Previous research has largely supported the dimension-specific pathways to psychopathology. For instance, threat in form of abuse, but not deprivation, was found to be associated with increased emotional reactivity and poor emotion regulation [7]. A more recent meta-analysis of 91 studies showed a stronger association of deprivation with working memory compared with threat (Hedges’ *g* = − 54 vs − 28) [15].

## Deprivation and adolescent psychopathology: potential mechanisms

DMAP theorizes that deprivation, due to diminished environmental inputs, is associated with psychopathology via deficits in cognitive and verbal abilities [8]. Children exposed to economic deprivation are found to have poorer language skills and executive functions [16], which in turn are linked to worse mental health [8, 17]. The link between deprivation and cognitive functioning has been supported by neuroimaging studies, showing reductions in cortical thickness and surface area across the cortex in children raised in socioeconomically deprived environment [18, 19]. Experimental studies also demonstrated that reducing poverty improved infant brain activity [10], indicating an enhanced neuroplasticity, which is associated with subsequent development of cognitive skills [10]. Taken together, evidence strongly suggests that suboptimal neural and cognitive development among children exposed to early deprivation is associated with later psychopathology [20, 21].

However, few longitudinal studies explicitly tested the mediating mechanisms between deprivation and later psychopathology. A study using structural equation modelling with prospective longitudinal data from the Growing Up in Scotland survey showed that childhood socioeconomic deprivation (characterised by income and subjective poverty) at age 1 was associated with higher levels of conduct problems at ages 4–6, mediated via cognitive ability at age 3 [22]. Another study based on data from the USA, which included information on children followed for over a decade, found that the association between deprivation (defined as a composite of household income and highest level of education) at ages 5–6 and externalising problems at age 17 was partially mediated via verbal abilities at age 14 [23].

## Threat and adolescent psychopathology: potential mechanisms

DMAP also postulates that emotion regulation is the key mechanism linking threat and later psychopathology [7, 8]. Emotion regulation is defined as the ability to monitor, evaluate, and modify emotions that arise in different situations [24]. Experience of threat is hypothesised to alter the development of cortical and subcortical circuits employed in fear learning and salience processing, which affects emotional processing, including attention and memory, emotional learning and reactivity, and regulation whilst facing negative emotional stimuli [24]. Threat adversities, such as physical punishment or harsh parenting, have been found to have a strong association with psychopathology, including internalising and externalising problems or psychological distress [8, 25]. Numerous studies found an association between threat and emotion regulation [8, 26–28], and between emotion regulation and psychopathology [29–33]; however, there is a dearth of longitudinal research explicitly assessing the mediating role of emotion regulation between threat and psychopathology. One study, based on data from the Longitudinal Studies on Child Abuse and Neglect in the USA, found that emotion regulation in the form of avoidant strategies may partially mediate the association between childhood exposure to threat and adolescent psychopathology, particularly internalising problems [34].

Noteworthy, threat exposures were shown to be uniquely associated with emotion regulation when deprivation was accounted for, and verbal abilities did not mediate the association between threat and internalising and externalising problems [23], which all suggests distinct developmental pathways between each dimension of adversity and psychopathology [8, 26, 34].

## Limitations of the current literature

The current literature is limited in several important ways. First, psychopathology was often reported by mothers [22, 23]. Mother or parent-reported measures tend to correlate poorly with child-reported measures [35]. Child-reported psychopathology is argued to provide a more accurate reflection of the child’s psychopathology, hence potentially showing differential associations with threat and deprivation [36, 37]. Second, a series of potential confounding factors, such as birth and infancy factors (e.g., birthweight or gestational age), family structure, and parental mental health were not adjusted for in previous studies. This may cause a strong threat to causal interpretations of the findings. Third, most studies used vulnerable samples, as opposed, to population-based ones. This may lead to overestimating the associations between threat and deprivation and psychopathology, compared to general population. Fourth, adolescent emotion regulation and psychopathology were measured concurrently, which failed to establish the temporal relationship and thus the relationship between these two factors is at risk of reverse causality [34]. Most importantly, no population-based study has attempted to examine the distinct developmental pathways between adversity and later psychopathology comprehensively within the same population.

## Current study

In the current study, we aimed to examine the extent to which associations between exposures to deprivation and threat in childhood and psychopathology, defined as psychological distress, in adolescence were mediated by cognitive ability and emotion regulation, respectively. As deprivation and threat are unlikely to ever be fully prevented (through primary prevention) and these experiences cannot be reverted afterwards, it is crucial to devise effective interventions or policies to prevent subsequent onset of psychopathology among already exposed individuals (secondary prevention). To build foundations for such interventions or policies, we need to understand the potential mediating mechanisms between exposures to deprivation and threat in childhood and onset of subsequent psychopathology. Using the Millennium Cohort Study (MCS), a large UK representative cohort, we examined the mediating mechanisms between exposures to deprivation and threat in early childhood (between ages 9 months and 5 years) and self-reported psychological distress at age 17 via cognitive ability at age 11 and emotion regulation at age 7, adjusting for a range of key confounders. We hypothesised that (1) the deprivation–psychological distress association will be mainly mediated by cognitive ability and less by emotion regulation; (2) the threat–psychological distress association will be mainly mediated by emotion regulation and less by cognitive ability.

## Methods

The protocol for this study was preregistered at https://osf.io/hnzmq/. This was done to ensure that our analyses were less affected by researcher bias.

### Population

The MCS is a longitudinal survey following a nationally representative, clustered and stratified sample of 19,000 children born in the UK in 2000–2002 [38]. The sample was drawn from all babies born between 1 September 2000 and 31 August 2001 in England and Wales and those born between 23 November 2000 and 11 January 2002 in Scotland and Northern Ireland. It was selected from a random sample of electoral wards, disproportionately stratified to ensure adequate representation of all four UK countries of deprived areas and areas with high concentrations of ethnic minority families. Seven waves of data have been collected at ages 9 months (2001), 3 years (2004), 5 years (2006), 7 years (2008), 11 years (2012), 14 years (2015), and 17 years (2018). Our target sample are singletons who were present at the seventh wave (age 17), resulting in a final sample of 10,709 participants. To ensure the representativeness of our sample to the UK general population, both sampling weights, which correct for MCS participants having unequal probabilities of selection due to the stratified cluster sample design, and inverse probability weighting, which calculates weights at the seventh wave to account for attrition, were used in all analyses. The Millennium Cohort Study data collections received full ethical approval from the National Health Service Multi-Centre Research and Ethics Committee at each wave [38].

### Measures

#### Deprivation

Several indicators were combined to derive the measure of deprivation (see section “Operationalisation of threat and deprivation” for details), including family income, area deprivation, lowest household academic qualification, parental occupational social class, parental unemployment, and housing tenure—all recorded when the child was between 9 months and 5 years, see Table 1 and eTable 1 for more details.

**Table 1:** Construction of exposure variables

	Time	Measurements
Construction of deprivation		
Family income	9 months, 3, and 5 years	Weekly household income was first equalized according to the Organisation for Economic Cooperation and Development household equivalence scale [54]. A count variable (range 0–3) was then derived indicating the number of times the equalized household income was below 60% of the UK median
Area deprivation	9 months, 3, and 5 years	The Indices of Multiple Deprivation was a weighted measure combining information on a low income, employment, health and disability, education and skills, housing and services, crime and environment at the Lower-layer Super Output Areas [55]. Each child was assigned a decile rank between 1 (most deprived) to 10 (least deprived) based on their postcode. A count variable (range 0–3) was then derived indicating the number of times the child was in the most deprived area (decile rank = 1)
Lowest household academic qualification	5 years	Parents reported their National Vocational Qualification (NVQ), which comprised six categories (NVQ 1–5, and ‘Other’) [56]. An index representing the lowest NVQ in the household was then created
Social class based on occupation	5 years	Parents’ occupation was classified as semi-routine and routine, lower supervisory and technical, small employers and self-employed, intermediate, and managerial and professional according to the National Statistics Socio-economic Classification [57]. An index was created indicating “both parents in routine/manual occupation”, “either parent in routine/manual occupation”, “neither parent in routine/manual occupation”. Social class of lone parents with manual occupation was categorised as “both parents in routine/manual occupation”
Parental unemployment	9 months, and 3 years	Unemployment status of both parents was reported by the mother, and a count variable (range 0–2) was derived indicating the number of times either parent was unemployed
Housing tenure	9 months, 3, and 5 years	A count variable (range 0–3) was derived indicating the number of reports of not owning a house
Construction of threat		
Interparental violence	9 months, 3, and 5 years	“Has your husband/wife ever used force on you for any reason?” was asked to a parent, with response options of “yes”, “no” or "don't know". A count indicator (range 0–3) was derived indicating how many times the child was exposed to interpersonal violence between ages 9 months and 5 years
Discipline practises	3 years	Two items from the Straus's Conflict Tactics Scale were asked to the mother: “how often do you do the following when the child is naughty 1) smack him/her/them; 2) shout at him/her/them?” (with response options: “once a month”, “once a week or more”, “daily”, “rarely”, “never”) [58] Two items from the Caldwell and Bradley’s Home Observation for Measurement of the Environment scale, which were used alongside other questions as part of the circumstances concerning cognitive testing, as completed by the interviewer: “Mother used physical restraint on child”, “Mother slapped or spanked child” (with response options: “yes”, “no”) [59]

#### Threat

The measure of threat was derived using individual items across several scales (see section “Operationalisation of threat and deprivation” for details), including parental interpersonal violence, and parental discipline practices, as reported by parents (smacking, shouting) and interviewers (using physical restraint, slapping or spanking) at age of 9 months, 3, and 5 years. The items reported by the interviewer were based on the observations during the cognitive testing, see Table 1 and eTable 1 for more details.

#### Cognitive ability

Cognitive ability at age 11 was operationalised as verbal knowledge and reasoning measured using the Verbal Similarities subscale of the British Ability Scales II. This scale was validated and standardised in a representative UK population of children aged 2–17 [39]. It has demonstrated a robust construct validity as a measure of cognitive ability and high test–retest reliability [39]. The subscale captured verbal reasoning ability, expressive language skills, including verbal fluency, vocabulary knowledge, general knowledge, abstract and logical thinking, ability to distinguish between essential and superficial features, and level of language stimulation [39]. Age-adjusted scores were adopted to facilitate potential comparisons with other studies, with a higher score representing greater cognitive ability. The subscale had a good internal consistency in our sample, with the Cronbach’s *α* of 0.84.

#### Emotion regulation

Emotion regulation was measured using a 5-item emotion dysregulation subscale of the Child Social Behavioural Questionnaire, reported by the parent when the child was 7 years [40]. Each item (e.g., “gets over excited”) was rated as “not true”, “somewhat true”, “certainly true”. The items were reversed when appropriate and summed, resulting in a variable ranging from 5 to 15, with a higher score representing better emotion regulation. The subscale had a good internal consistency in our sample, with the Cronbach’s *α* of 0.70.

#### Psychopathology

Psychopathology at age 17 was defined as psychological distress. Psychological distress was self-reported using the Kessler 6 (K6) scale, with a sensitivity of 0.36 and a specificity of 0.96 to detect serious mental illness [41]. It consists of six questions about depressive and anxiety symptoms that a person has experienced in the last 30 days (e.g., worthless, nervous, hopeless). The response scale ranges from “all of the time” (4) to “none of the time” (0). The higher score, obtained by summing up the items, indicates greater psychological distress. The Cronbach’s *α* of K6 scale in our study was 0.86.

#### Confounding

Potential confounders were identified a priori, as variables that could influence the exposure–mediator, mediator–outcome and/or exposure–outcome relationships [42]. First, lead authors selected potential confounding factors based on recently published longitudinal studies examining the pathways of the DMAP model or the specific associations between each exposure, mediator and outcome. For instance, we reviewed studies of the link between deprivation and psychopathology [43], deprivation and cognition [8], threat and psychopathology [34], threat and emotion regulation [41]. Then, lead authors operationalised them with the available indicators, and then discussed them with the other members of the study team.

The potentially confounding factors used in our study can be broadly categorised chronologically as baseline confounders (that confound the associations between exposure, mediator and outcome) and intermediate confounders (that confound the mediator–outcome association).

The baseline confounders (exposure–mediator–outcome) included: sex, ethnicity, coming from a lone parent household, number of siblings of study child at birth, maternal age at birth, planned or unplanned pregnancy, maternal and paternal psychological distress measured using the Kessler K6 scale administered when the study child was 3 years, whether the mother smoked during pregnancy, whether the father smoked during pregnancy, and whether the mother drank alcohol during pregnancy. The intermediate confounders (mediator–outcome) included: birthweight of the child in kilograms, gestational age, month of birth (as a reflection of relative age throughout childhood), and whether the child was breastfed. The intermediate confounders provide proxy information on infant’s health and are typically socioeconomically stratified [44], but they are unlikely causes of threat or deprivation. In rare cases, an infant with poor birth characteristics (e.g., low birthweight) could develop long-lasting health problems that limit parental capacity to earn living, potentially leading to economic deprivation. However, variables, such as birth weight and gestational age, could not be considered valid indicators of long-term health.

Parental smoking and drinking during pregnancy were specified in the study protocol (available at: https://osf.io/hnzmq/) as mediator–outcome confounding; however, after further elaboration, we assumed that these factors are also likely to be associated with subsequent deprivation and threat (exposures).

### Analysis

The analysis code is available online (https://osf.io/hnzmq).

#### Operationalisation of threat and deprivation

We conducted confirmatory factor analysis (CFA) to validate the underlying factor structure of the measurement model [45]. The fit of the model was determined using four fit indices at given thresholds—root mean squared error of approximation (RMSEA < 0.08), comparative fit index (CFI > 0.90), Tucker–Lewis index (TLI > 0.90), and standardized root mean squared residual (SRMR < 0.08) [46, 47]. After fitting the model, continuous latent scores reflecting threat and deprivation for each cohort member were obtained with higher scores representing higher levels of threat and deprivation. As causal mediation analysis under counterfactual framework requires two levels of the exposure for comparison to calculate natural direct/indirect effect, we binarized the latent score of deprivation and threat. Despite the potential drawbacks of binarization (e.g., loss of information, reduced power, increased risk for type II error), our decision was determined by the constraints of our methodological approach and a greater interpretability of the findings. Since the histogram of latent scores did not give a clear cutoff (eFigure 1), a cutoff at its 75th percentile was chosen as a common practice for dichotomising risk factors in the literature [48, 49].

#### Studying mechanisms

**Fig. 1:**
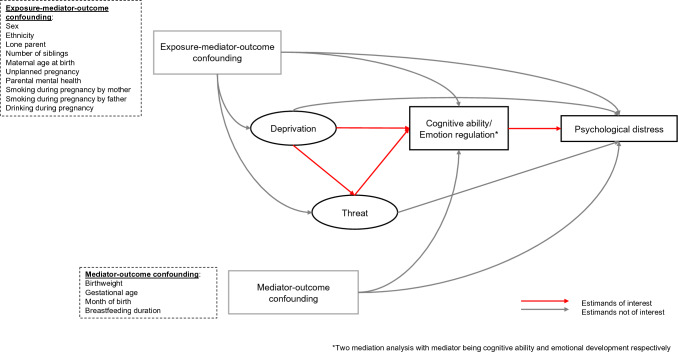
Causal diagram for estimands of interest when deprivation is the exposure

**Fig. 2:**
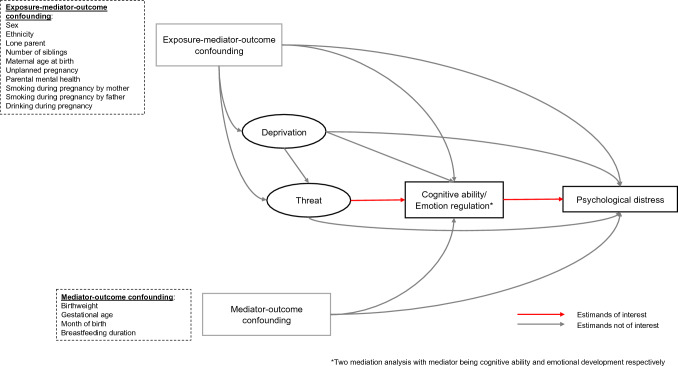
Causal diagram for estimands of interest when threat is the exposure

We estimated natural indirect effects (NIE) by conducting causal mediation analysis under the counterfactual framework, which can deal with intermediate confounding factors affected by exposure (i.e., exposure to threat when examining mediation mechanism between exposure to deprivation and psychological distress, as shown in Fig. 1). We present Directed Acyclic Graphs for the relationships between deprivation, threat, cognitive ability, emotion regulation, and psychological distress in Figs. 1 and 2. Noteworthy, we assumed that there is an arrow from deprivation to threat, as previous evidence suggests that economic deprivation is likely to be an antecedent of various forms of childhood adversity [50]. Two main estimands are of interest in the current study (see Table 2 for details): NIE of deprivation via cognitive ability/emotion regulation on psychological distress (Fig. 1), and NIE of threat via cognitive ability/emotion regulation on psychological distress (Fig. 2). Estimation of the two estimands has been articulated in the proposal register at https://osf.io/hnzmq/. Namely, randomized interventional analogues of the NIE (rNIE) was estimated for the NIE of deprivation on psychological distress via cognitive ability/emotion regulation, as threat acts as an intermediate confounding factor (see Fig. 1), which is defined as a confounder of the association between cognitive ability/emotion regulation and psychological distress that is influenced by deprivation [50]. Meanwhile, NIE was estimated directly for the NIE of threat on psychological distress via cognitive ability/emotion regulation given the absence of intermediate confounding factor.

**Table 2:** Description of estimands of interest

Estimand of interest	Definition	Confounding to account for
Deprivation → cognitive ability/emotion regulation → psychological distress	rNIE of deprivation on psychopathology through cognitive ability/emotion regulation	Exposure–mediator–outcome confounding: Sex, ethnicity, lone parent, number of siblings, maternal age at birth, unplanned pregnancy, parental mental health, smoking during pregnancy by mother, smoking during pregnancy by father, drinking during pregnancy Mediator–outcome confounding: Birthweight, gestational age, month of birth, breastfeeding duration *Exposure-induced confounding: Threat
Threat → cognitive ability/emotion regulation → psychological distress	NIE of threat on psychopathology through cognitive ability/emotion regulation	Exposure–mediator–outcome confounding: Sex, ethnicity, lone parent, number of siblings, maternal age at birth, unplanned pregnancy, parental mental health, smoking during pregnancy by mother, smoking during pregnancy by father, drinking during pregnancy, deprivation Mediator–outcome-confounding: Birthweight, gestational age, month of birth, breastfeeding duration

The strength of applying causal mediation analysis under the counterfactual framework in our study is that it enables the estimation of indirect effects when there is intermediate confounding factor (the case for the deprivation pathway) and it has policy relevant implications [51]. Apart from the assumption of no unmeasured confounding factors given those listed in Fig. 1, other assumptions, such as positivity, consistency and no interference, are still needed for our estimates to be interpreted causally [51, 52].

CMAverse package in R was employed to conduct the causal mediation analysis. A regression-based approach was applied to examine mediating mechanism between exposure to threat and psychological distress, and g-formula approach was applied to examine mediating mechanism between exposure to deprivation and psychological distress. In both cases, direct counterfactual imputation estimation was adopted, and standard errors of all estimands of interests were estimated via bootstrapping. Missing data were dealt with using multiple imputation to minimise the impact of biased attrition and non-response on the estimates. The details on missing data strategy can be found in eText 1, including information on missing data (eTable 2) and predictors of missingness (eTable 3). All analyses were conducted in Stata 17.0 and R 4.1.0.

## Results

### Descriptive information

The mean level of psychological symptoms was 7.28 (standard deviation 4.93) in the study sample. The mean scores were 59.14 (sd 10.03) on cognitive ability measure (range 20–80), and 11.51 (sd 2.36) on emotion regulation (range 5–15). Most of the sample constituted white participants (81.7% white vs 18.3% non-white), with nearly equal distribution of females (50.2%) and males (49.8%), see Table 3 for more detailed descriptive information about participants.

**Table 3:** Descriptive information about the studied variables—among those with complete measure of psychological distress (*n* = 10,709)

Variable	*N* total	Mean	SD
Outcome			
Psychological distress^a^	9882	7.28	4.93
Mediators			
Cognitive ability^b^	9893	59.14	10.03
Emotion regulation^c^	9083	11.51	2.36
Exposures			
Deprivation	5454		
High (top 25%)		4092	75.0
Low (bottom 75%)		1362	25.0
Threat	5561		
High (top 25%)		4231	76.1
Low (bottom 75%)		1330	23.9
Baseline confounding factors			
Child’s sex	10,331		
Female		5190	50.2
Male		5141	49.8
Child's ethnicity	10,310		
White		8426	81.7
Non-white		1884	18.3
Unplanned pregnancy	10,301		
Pregnancy was a surprise		4352	42.2
Planning to get pregnant		5949	57.8
Lone parent at birth	10,328		
No (reference)		10,043	97.2
Yes		285	2.8
Maternal age at birth	10,331		
12–19		400	3.9
20–29		3139	30.4
30–39		5751	55.7
≥ 40		1041	10.1
Number of siblings	10,331		
0		4380	42.4
1		3585	34.7
2		1560	15.1
≥ 3		806	7.8
Mother’s mental health^d^	9473	3.09	3.70
Father’s mental health^d^	7046	2.94	3.26
Smoking during pregnancy by mother	10,301		
No (reference)		7093	68.9
Yes		3208	31.1
Smoking during pregnancy by father	7918		
No (reference)		5144	65.0
Yes		2774	35.0
Drinking during pregnancy by either parent	10,314		
No (reference)		7171	69.5
Yes		3143	30.5
Subsequent confounding factors			
Birthweight	10,307	3.37	0.58
Gestational age	10,208	276.28	13.53
Month of birth	10,331	6.69	3.50
Child was breastfed	10,316		
No (reference)		2820	27.3
Yes		7496	72.7

*SD* standard deviation

^a^Higher score indicates higher psychological distress

^b^Higher score indicates higher cognitive ability

^c^Higher score indicates higher emotional regulation

^d^Higher score indicates lower mental health

### Measurement model of exposures

Confirmatory factor analysis indicated that the measurement model of deprivation had a strong fit (RMSEA = 0.046; CFI = 0.985; TLI = 0.975; SRMR = 0.024) (see Table 4). The model including all five items measuring threat (reported by parents: interpersonal violence, smacking, shouting; reported by interviewers: using physical restraint, slapping or spanking) had a poor fit (RMSEA = 0.092; CFI = 0.806; TLI = 0.612; SRMR = 0.053). As post-hoc adjustment of the pre-registered analysis, we removed an indicator of slapping or spanking due to low prevalence (n = 56; 0.6%). This resulted in improvement of the measurement model of threat (RMSEA = 0.000; CFI = 1.000; TLI = 1.000; SRMR = 0.003). As a sensitivity check, we compared latent scores derived from both models, which had a perfect correlation (r = 0.999).

**Table 4:** Statistics indicating the fit of measurement models of deprivation and threat

Exposure	Components included	Fit statistic	
Deprivation (*n* = 5454)^a^	Family income, area deprivation, household academic qualification, social class based on occupation, parental unemployment, housing tenure	RMSEA	0.046 (95% CI 0.039, 0.054)
		CFI	0.985
		TLI	0.975
		SRMR	0.024
Threat (*n* = 5561)^a^	Interparental violence, discipline practises (i.e., smacking, shouting, physical restraint) *Slapping or spanking was excluded due to low prevalence (*n* = 56; 0.6%)	RMSEA	0.000 (95% CI 0.000, 0.019)
		CFI	1.000
		TLI	1.000
		SRMR	0.003

*RMSEA* Root mean squared error of approximation, *CFI* Comparative fit index, *TLI* Tucker–Lewis index, *SRMR* Standardized root mean squared residual

^a^The sample size includes participants without missing data on any of the components within each exposure

### Associations between study variables

We do not present coefficients between confounding factors and psychological distress to avoid their over-interpretation; however, a correlation table among all study variables was provided (see eTable 4).

There was no evidence for mean differences in psychological distress across levels of deprivation (high vs low 0.21; 95% CI − 1.32, 1.74) or threat (high vs low − 0.07; 95% CI − 1.83, 1.68) in the adjusted model (see Table 5 for both unadjusted and adjusted estimates).

**Table 5:** Association between exposures and psychopathology and between mediators and psychopathology—estimates using imputed data (*n* = 10,709)

Type of association	Unadjusted model		Adjusted model	
	*b*	95% CI	*b*	95% CI
Exposures–psychological distress associations				
Deprivation (high vs low)	0.46	(− 0.70, 1.62)	0.21	(− 1.32, 1.74)
Threat (high vs low)	− 0.17	(− 2.00, 1.66)	− 0.07	(− 1.83, 1.68)
Mediators–psychological distress associations				
Cognitive ability	0.02	(− 0.02, 0.06)	0.04	(0.00, 0.07)
Emotion regulation	− 0.13	(− 0.27, 0.01)	− 0.15	(− 0.28, − 0.02)
Exposures–cognitive ability associations				
Deprivation (high vs low)	− 5.72	(− 7.40, − 4.05)	− 3.91	(− 5.64, − 2.17)
Threat (high vs low)	− 1.26	(− 2.26, − 0.27)	− 1.41	(− 2.40, − 0.41)
Exposures–emotion regulation associations				
Deprivation (high vs low)	− 1.00	(− 1.32, − 0.69)	− 0.32	(− 0.69, 0.05)
Threat (high vs low)	− 1.04	(− 1.25, − 0.83)	− 0.82	(− 1.05, − 0.59)

Adjusted model controlled for sex, ethnicity, lone parent, number of siblings, maternal age at birth, unplanned pregnancy, parental mental health, smoking during pregnancy by mother, smoking during pregnancy by father, drinking during pregnancy, deprivation (unless deprivation is the exposure); for mediators–psychopathology associations, following factors were further adjusted for: birthweight, gestational age, month of birth, whether the child was breastfed

*B* beta coefficient (unstandardised), *95% CI* 95% confidence interval

Higher cognitive ability was associated with higher levels of psychological distress (b = 0.04; 95% CI 0.00, 0.07), whereas greater emotion regulation was linked with lower symptoms of psychological distress (b = -0.15; 95% CI − 0.28, − 0.02) in the adjusted models.

Most deprived participants (top 25%) had on average 3.91 (95% CI − 5.64, − 2.17) lower cognitive ability score than those in low deprivation, in the adjusted model. The mean difference was more modest for the threat variable, with the top 25% having 1.41 (95% CI − 2.40, − 0.41) lower cognitive ability score.

Most deprived participants had on average 0.32 (95% CI − 0.69, 0.05) lower score on emotion regulation, in the adjusted model. The mean difference was greater for threat variable, with those in the high threat group having 0.82 (95% CI − 1.05, − 0.59) lower score compared with those in the low threat group.

### Mediation analysis

As in the regression analysis, we found no total effect of either exposure to deprivation or threat on psychological distress (see Table 6 for results of mediation analysis). However, the indirect effects of four potential mediating pathways were significant, with deprivation on psychological distress via cognitive ability being -0.11 (95% CI − 0.20, − 0.05) and threat on psychological distress via emotion regulation being 0.09 (95% CI 0.03, 0.15), whereas the indirect effect of deprivation on psychological distress via emotion regulation was 0.03 (95% CI 0.02, 0.12) and threat on psychological distress via cognitive ability was − 0.04 (95% CI − 0.07, − 0.01).

**Table 6:** Direct, indirect and total effect between exposures and psychological distress

		Direct effect	Indirect effect	Total effect
Deprivation pathway	Via cognitive ability	0.40 (− 0.16, 0.84)	− 0.11 (− 0.20, − 0.05)	0.29 (− 0.24, 0.75)
	Via emotion regulation	0.26 (− 0.30, 0.70)	0.03 (0.02, 0.12)	0.29 (− 0.24, 0.76)
Threat pathway	Via cognitive ability	− 0.13 (− 0.41, 0.29)	− 0.04 (− 0.07, − 0.01)	− 0.16 (− 0.45, 0.27)
	Via emotion regulation	− 0.25 (− 0.55, 0.20)	0.09 (0.03, 0.15)	− 0.16 (− 0.45, 0.27)

Mediator was modelled separately for each pathway

### Post-hoc analyses

As post-hoc analyses, we assessed the total effect of deprivation and threat, defined as continuous variables, on psychological distress. As in the original analysis, we found very weak evidence for the relationship (deprivation: b = 0.02; 95% CI − 0.33, 0.37; threat: b = − 1.33; 95% CI − 5.30, 2.63). In addition, we examined the extent to which any of the individual components of either deprivation or threat was associated with psychological distress, finding very weak evidence for such associations (see eTable 5).

## Discussion

### Key findings

We found no evidence for the hypothesised association between deprivation and threat in childhood and psychological distress at age 17 in the MCS. This has made the key objective of our study somewhat redundant, as we were interested in informing (secondary) prevention of psychological distress among individuals already exposed to deprivation or threat. We did, however, find statistically significant evidence for indirect effects of both deprivation and threat on psychological distress, which partially supported our hypotheses. Finding indirect effect in the absence of the total effect is not unusual in the literature [53, 54], and we go on to discuss potential reasons for this, along with providing speculative explanations of why total effect was not detected in the first place.

### Interpretation and implications of findings

Both exposures—deprivation and threat—were not found to be associated with psychological distress in our study. This is at odds with a vast literature, using range of psychopathology outcomes, such as hyperactivity, emotional symptoms, conduct problems, peer problems, prosocial behaviour, psychological distress or depressive disorders [8, 22, 23, 25, 26, 34, 43, 55, 56]. One potential explanation for these unexpected findings is that most of the literature relies on parent-reported measures of psychopathology [8, 9, 22, 23, 26, 34, 43, 55, 56], whereas symptoms were reported by children themselves in our study.

For instance, previous studies tend to find relatively modest socioeconomic inequality in child-reported psychological distress but larger socioeconomic inequality when the symptoms are reported by parents [43, 55–57]. One study using MCS found that having below 60% of median income at age 14 was associated with minimally greater child-reported psychological distress at age 17 (mean of 0.22, range 0–24) [58]. Likewise, income had a weak association with child-reported psychological distress measured at age 14, and only among girls [35]. Previous research based on the MCS found that threat-related variables, such as inter-parental use of force, parental discord, harsh parenting, and physical punishment were all associated with parent-reported internalising and externalising problems at ages 3–14 years [59]. However, when the psychological distress was reported by children at age 17, in the Avon Longitudinal Study of Parents and Children, a threat-related variable (violence between parents) was not associated with depression [60]. Reporting bias appears to exist not only for the associations between deprivation and threat and psychological distress but also between cognitive ability and psychological distress. Cognitive ability, defined as verbal knowledge and reasoning in MCS, was negatively associated with psychological distress when symptoms were reported by parents, but positively when self-reported by children (as also found in our study) [35].

Another explanation for the null finding is that both deprivation and threat are more strongly associated with behavioural problems in adolescence, including illicit drug use and smoking, whereas the link with depressive symptoms develops later in the life course [1, 57, 60, 61]. Some of the previous studies reported strong associations between deprivation and threat-related variables and psychological distress reported by children in their adolescence [25, 61]. However, these were based on older British birth cohorts, including those born in 1958 and 1970. Hence, it is possible that the link between these exposures and psychological distress has weakened over time due to changing social context, for instance, policies aiming to improve life opportunities for children from particularly vulnerable background. However, this may be overoptimistic outlook and further studies are warranted to explicitly test this hypothesis. Importantly, we do not suggest that policies improving life chances of deprived individuals are not needed. There is also a possibility that threat indicators do not adequately capture perception of threat by the child, as they were reported by parents and interviewers. Unfortunately, child-reported measure of threat was unavailable. We considered including bullying into our definition of threat, as its frequency was provided by children. However, bullying was deemed to be too broad a concept, as it may include exclusion and gossiping that are not considered threat components.

Despite the lack of total effect of exposure to deprivation and threat on psychological distress, we proceeded to conduct the causal mediation analysis as previously registered, because of the hypothesised mediating pathways and the fact that the indirect effect test has more power to be detected than the total effect [53, 54]. Indeed, significant indirect effects were detected, while total and direct effects were null. Thus, as discussed above, we do not have enough evidence to show that exposure to deprivation or threat could lead to psychological distress. However, the significant indirect effect indicates that if exposure to deprivation or threat did lead to psychological distress as proven by previous studies, our a priori hypotheses hold in that the deprivation–psychological distress pathway was mainly mediated by cognitive ability and less by emotion regulation (− 0.11 vs 0.03), and the threat–psychological distress pathway was mainly mediated by emotion regulation and less by cognitive ability (0.09 vs − 0.04). However, it should still be borne in mind that the statistically significant indirect effects were so small (psychological distress symptoms range from 0 to 24) that it may not have practical implications for policy making and design of intervention.

### Strengths and limitations

The major strengths of our study include using a prospective, largely UK-representative birth cohort, with 17 years of follow-up. Our study takes advantage of the rich information collected not only from study participants, but also their parents. In addition, we preregistered our study, which helped to reduce researcher bias and control Type I error [62].

Our study is also subject to several limitations. First, our sample suffers from missing information due to attrition and non-response, which appears to be greater in more socioeconomically disadvantaged populations (see eTables 2 and 3). This can potentially lead to underestimation of the association between deprivation and psychological distress. However, this limitation was mitigated, at least to some extent, by inverse probability weighting and multiple imputation, which allowed us to minimise bias in estimates by taking advantage of rich information available in the cohort [63].

Second, we only focused on economic deprivation due to unavailability of information about cognitive, emotional or social deprivation in this cohort. Economic deprivation captures a single component of deprivation and could be considered as a partial proxy for the wider conceptualisation of deprivation in the DMAP model. There have been concerns about using such a narrow definition of deprivation, as it may be difficult to disentangle the role of poverty in threat and social/cognitive deprivation [64, 65]. In our study, we assumed that deprivation precedes threat, hence adjusting for deprivation when estimating the impact of threat along the causal chain. This was due to the relatively consistent evidence showing that early life deprivation often underpins other forms of deprivation [66], tends to cluster with a range of adversities [50] and is associated with later psychopathology in childhood and adolescence [43, 55, 56, 61].

Third, despite adjusting for a range of potentially confounding factors, there is still a possibility that our estimates suffer from residual confounding bias, for instance, due to genetic factors influencing both experiences of threat or deprivation and psychopathology. In a similar vein, our decision to classify the confounding factors as exposure–mediator–outcome or mediator–outcome is to some extent based on subjective judgement. For instance, as pointed out by one of the reviewers, socioeconomic circumstances after birth are likely to be highly correlated with socioeconomic circumstances before or during birth. Hence, birth factors, such as birthweight or gestational age could be classified as potential mediators of the relationship between deprivation and psychological distress.

Fourth, our measures of threat were assessed via self-reports, which can be influenced by social desirability and norms [67]. Hence, they can be underreported and potentially bias the results towards the null. An analysis using additional, more objective measures of threat, for instance, through intense observations, or based on child reports would provide a more holistic picture of potential impact of this exposure on psychopathology.

Finally, psychological distress was also self-reported using a questionnaire, rather than being ascertained by a clinical interview. The Kessler-6 scale asks about current levels of symptoms which may be prone to influences by recent life circumstances, as opposed to long-term aspects of psychological distress that are more likely to be influenced by childhood experiences.

## Conclusion

We found no evidence for the hypothesised association between exposure to deprivation and threat, or any of their individual components, in childhood and psychological distress in adolescence but did find significant indirect effects via cognitive ability and emotion regulation. We speculated on several potential reasons for these unexpected findings, which were largely at odds with existing studies. These included potential reporting bias, as most of the studies relied on parent-reports of psychological distress, as opposed to child-reports used in our analysis.

### Supplementary Information

Below is the link to the electronic supplementary material.

Supplementary file1 (DOCX 365 KB)

## Data Availability

MCS is deposited with the UK Data Service at the University of Essex.
