# Supplementary material for: Mediating mechanisms of the relationship between exposure to deprivation and threat during childhood and adolescent psychopathology: evidence from the Millennium Cohort Study
Source: Eur Child Adolesc Psychiatry. 2023 Sep 7;33(6):1907–20. doi: 10.1007/s00787-023-02289-3 (PMC11211201; doi:10.1007/s00787-023-02289-3)
Supplement: Supplementary file 1 — Supplementary file1 (DOCX 365 KB) [file 787_2023_2289_MOESM1_ESM.docx]

**Mediating mechanisms of the relationship between exposure to deprivation and threat during childhood and adolescent psychopathology: evidence from the Millennium Cohort Study**

Ke Ning*^1^, Dawid Gondek*^ⴕ2,3^, Snehal M. Pinto Pereira^4^, Rebecca E. Lacey^2^

^1^School of Public Health, LKS Faculty of Medicine, The University of Hong Kong, Hong Kong

^2^Research Department of Epidemiology and Public Health, University College London, London, England

^3^Swiss Centre of Expertise in Life Course Research (LIVES), University of Lausanne, Lausanne, Switzerland

^4^Division of Surgery and Interventional Science, University College London, London, England

*Joint lead authors

^ⴕ^ Corresponding author:

Dawid Gondek [dawid.gondek@unil.ch](mailto:dawid.gondek@unil.ch)

eText 1 Missing data strategy.

Missing information ranged from 3.5% on child’s sex, number of siblings, maternal age at birth, and month of birth to 48.3% on threat (see eTable 2). Having missing information on any of the study variables was predicted by higher psychological distress and deprivation, lower cognitive ability and emotional regulation, and being non-white (see eTable 3).

For the regression-based analyses, the missing data were also imputed using Multiple Imputation by Chained Equations Algorithm (MICE), with n=50 imputed datasets, due to the non-monotone pattern of missing values, and due to its ability to accommodate various types of variables in the imputation model, including continuous and categorical ones. This approach uses a series of univariate conditional imputation models to impute missing data.^1^ The multiple imputation works under the assumption that the data are missing at random (MAR).^2, 3^ The MAR mechanism, which is largely untestable, implies that systematic differences between the missing and the observed values can be explained by observed data.^2^ The strength of multiple imputation is that it tends to perform better than complete case analysis in most missing data scenarios, particularly when the amount of missing data is large.^4^

The imputation model included all variables included in analytical model (the outcome, mediators, exposures and potential confounding). In addition, we included an alternative measure of psychopathology (the Strengths and Difficulties Questionnaire), collected at age 17 from the cohort member. As this measure was associated with the study variables and missingness, it was likely to improve predictive power of the imputation model.^5, 6^ It is recommended to impute variables in the form.^7, 8^ Hence, we conducted three separate imputation models, including three versions of the threat and deprivation variables used in the study, as binary, continuous, and their individual components.

The mediation analysis applied CMAverse package, which by default conducts MICE to impute missing data. All variables including exposure, mediator, outcome and baseline/intermediate confounding factors in the main analysis were included at the imputation stage, and 20 datasets were imputed due to constrained calculation power.

| eTable 1 Distribution of items to construct deprivation and threat | |  |  | |
| --- | --- | --- | --- | --- |
| Items for deprivation | |  | Items for threat | |
| Times of household income below deprivation threshold at 9 months, 3 and 5 years old | |  | Times of exposing to interpersonal violence at 9 months, 3 and 5 years old | |
| 0 | 5176 (58.1%) |  | 0 | 5289 (81.9%) |
| 1 | 1272 (14.3%) |  | 1 | 645 (10.0%) |
| 2 | 1009 (11.3%) |  | 2 | 309 (4.8%) |
| 3 | 1459 (16.4%) |  | 3 | 217 (3.4%) |
| Times of child from the most deprived area at 9 months, 3 and 5 years old | |  | Frequency of smacking child at age 3 | |
| 0 | 4688 (80.6%) |  | Never | 2862 (33.4%) |
| 1 | 223 (3.8%) |  | Rarely | 4461 (52.0%) |
| 2 | 160 (2.7%) |  | Once a month | 445 (5.2%) |
| 3 | 748 (12.9%) |  | Once a week or more | 728 (8.5%) |
| Lowest household academic qualification at age 5 | |  | Daily | 80 (0.9%) |
| NVQ1 | 1030 (10.3%) |  | Frequency of shouting at child at age 3 | |
| NVQ2 | 3604 (35.9%) |  | Never | 270 (3.2%) |
| NVQ3 | 1562 (15.6%) |  | Rarely | 2727 (31.9%) |
| NVQ4 | 2562 (25.5%) |  | Once a month | 703 (8.2%) |
| NVQ5 | 217 (2.2%) |  | Once a week or more | 3368 (39.3%) |
| Others | 1069 (10.6%) |  | Daily | 1492 (17.4%) |
| Parental occupational social class at age 7 | |  | Mother using physical restraint on child at age 3 | |
| Neither parent in manual occupation | 5471 (57.0%) |  | No | 8826 (96.0%) |
| Either parent in manual occupation | 1969 (20.5%) |  | Yes | 369 (4.0%) |
| Both parents in manual occupation | 2155 (22.5%) |  | Mother slapped or spanked child at age 3 | |
| Times of either parental in unemployment at 9 months and 3 years old | |  | No | 9139 (99.4%) |
| 0 | 4022 (42.7%) |  | Yes | 56 (0.6%) |
| 1 | 1924 (20.4%) |  |  |  |
| 2 | 3465 (36.8%) |  |  |  |
| Times of not reporting owing a house at 9 months, 3 and 5 years old | |  |  |  |
| 0 | 5607 (62.7%) |  |  |  |
| 1 | 729 (8.1%) |  |  |  |
| 2 | 478 (5.3%) |  |  |  |
| 3 | 2131 (23.8%) |  |  |  |

eFigure 1 Histogram of latent scores of deprivation and threat


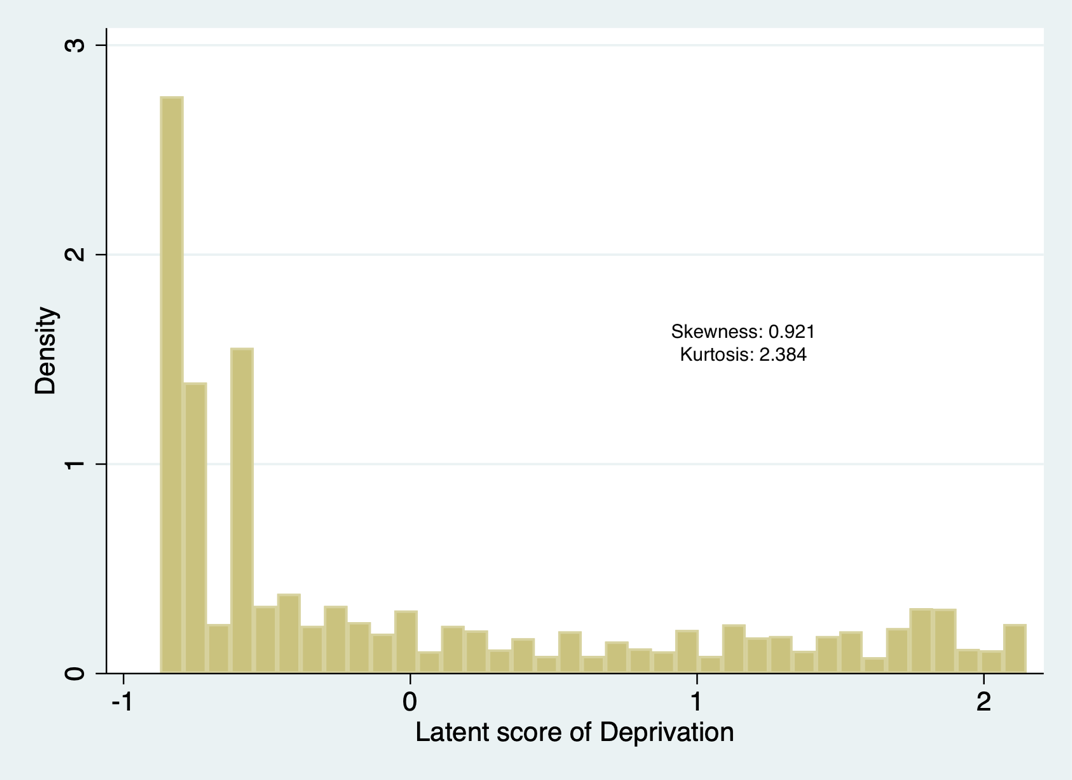

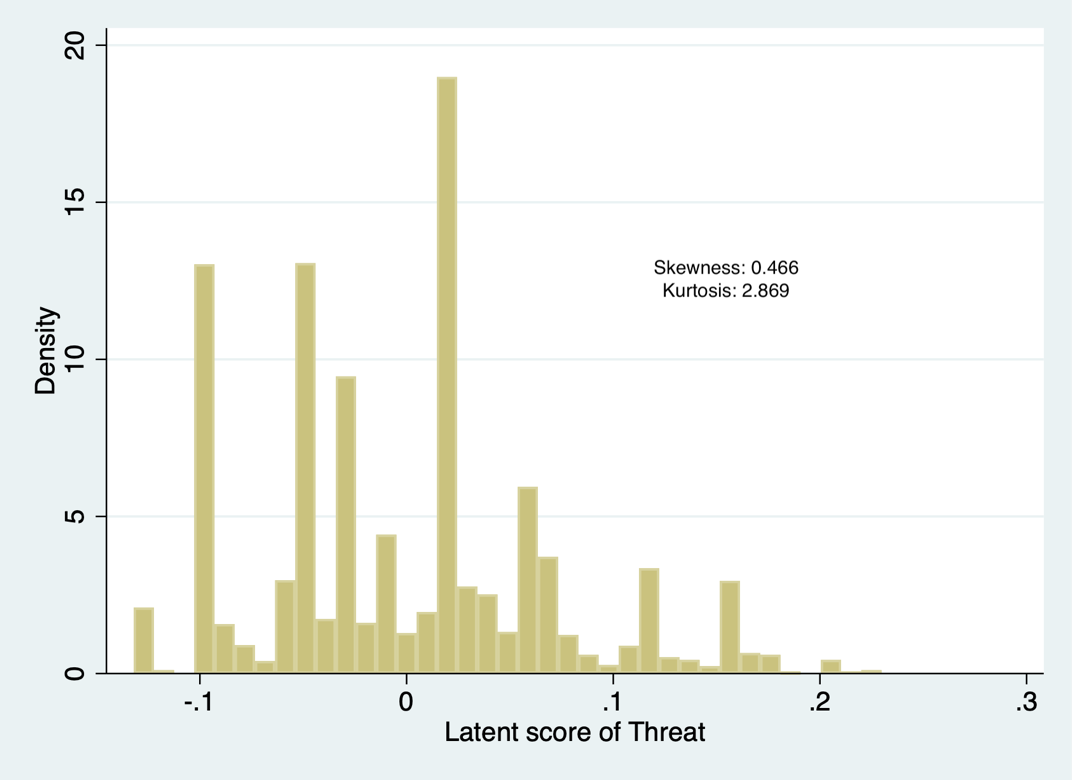


| eTable 2. The proportion of missing information in each variable (n=10,709). | | |
| --- | --- | --- |
| Variable | N missing | % missing |
| *Outcome* |  |  |
| Psychological distress | 827 | 7.7 |
|  |  |  |
| *Exposures* |  |  |
| Threat | 5,065 | 47.3 |
| Deprivation | 5,173 | 48.3 |
|  |  |  |
| *Mediators* |  |  |
| Cognitive ability | 816 | 7.6 |
| Emotion regulation | 1,626 | 15.2 |
|  |  |  |
| *Confounding* |  |  |
| Child’s sex | 378 | 3.5 |
| Child’s ethnicity | 399 | 3.7 |
| Lone parent at birth | 381 | 3.6 |
| Number of siblings | 378 | 3.5 |
| Maternal age at birth | 378 | 3.5 |
| Unplanned pregnancy | 408 | 3.8 |
| Mother’s mental health | 1,236 | 11.5 |
| Father’s mental health | 3,663 | 34.2 |
| Smoking during pregnancy by mother | 408 | 3.8 |
| Smoking during pregnancy by father | 2,791 | 26.1 |
| Drinking during pregnancy by either parent | 395 | 3.7 |
| Birthweight | 402 | 3.8 |
| Gestational age | 501 | 4.7 |
| Month of birth | 378 | 3.5 |
| Child was breastfed | 393 | 3.7 |

| eTable 3. Predictors of having missing information on any exposure in the study sample (n=10,709). | | | |
| --- | --- | --- | --- |
| Exposures | Risk ratio | 95% CI | |
| Psychological distress | 1.00 | 1.00 | 1.01 |
| Threat | 0.83 | 0.63 | 1.10 |
| Deprivation | 1.41 | 1.40 | 1.43 |
| Cognitive ability | 0.99 | 0.98 | 0.99 |
| Emotion regulation | 0.93 | 0.92 | 0.94 |
| Child’s ethnicity |  |  |  |
| White (reference) | - | - | - |
| Non-white | 1.91 | 1.84 | 1.98 |
| Child’s sex |  |  |  |
| Female (reference) | - | - | - |
| Male | 0.97 | 0.93 | 1.01 |
| *Note.* 95% CI = 95% confidence interval. | | | |

eTable 4. Correlation table of study variables

|  | Deprivation | Threat | Cog ability | Emo reg | Mental stress | Male | Non-white | Maternal age | Planned preg | Maternal K6 | Paternal K6 | Lone parent | No. of siblings | Mom drank | Mom smoked | Dad smoked | Birth weight | Breastfed | Gestage |
| --- | --- | --- | --- | --- | --- | --- | --- | --- | --- | --- | --- | --- | --- | --- | --- | --- | --- | --- | --- |
| Deprivation | 1.00 |  |  |  |  |  |  |  |  |  |  |  |  |  |  |  |  |  |  |
| Threat | -0.01 | 1.00 |  |  |  |  |  |  |  |  |  |  |  |  |  |  |  |  |  |
| Cognitive ability | -0.29* | -0.05* | 1.00 |  |  |  |  |  |  |  |  |  |  |  |  |  |  |  |  |
| Emotion regulation | -0.23* | -0.23* | 0.17* | 1.00 |  |  |  |  |  |  |  |  |  |  |  |  |  |  |  |
| Mental distress | 0.03* | -0.02 | 0.03* | -0.07* | 1.00 |  |  |  |  |  |  |  |  |  |  |  |  |  |  |
| Male | 0.00 | 0.13* | 0.05* | -0.13* | -0.28* | 1.00 |  |  |  |  |  |  |  |  |  |  |  |  |  |
| Non-white | 0.44* | -0.06* | -0.14* | -0.12* | -0.09* | 0.00 | 1.00 |  |  |  |  |  |  |  |  |  |  |  |  |
| Maternal age | -0.33* | -0.10* | 0.15* | 0.20* | -0.01* | 0.01 | -0.03* | 1.00 |  |  |  |  |  |  |  |  |  |  |  |
| Planned preg* | -0.37* | -0.01 | 0.13* | 0.16* | -0.03* | 0.00 | -0.16* | 0.31* | 1.00 |  |  |  |  |  |  |  |  |  |  |
| Maternal K6 | 0.24* | 0.10* | -0.09* | -0.26* | 0.09* | 0.02 | 0.15* | -0.08* | -0.17* | 1.00 |  |  |  |  |  |  |  |  |  |
| Paternal K6 | 0.20* | 0.03 | -0.03* | -0.12* | 0.09* | 0.03* | 0.12* | -0.02 | -0.06* | 0.19* | 1.00 |  |  |  |  |  |  |  |  |
| Lone parent | 0.29* | 0.04 | -0.08* | -0.18* | 0.08* | 0.02 | 0.07 | -0.13* | -0.28* | 0.13* | 0.07* | 1.00 |  |  |  |  |  |  |  |
| No. of siblings | 0.24* | -0.05* | -0.14* | 0.05* | -0.01 | 0.00 | 0.16* | 0.33* | -0.12* | 0.08* | 0.05* | -0.01 | 1.00 |  |  |  |  |  |  |
| Mom drank | -0.29* | 0.08* | 0.12* | 0.04* | 0.03* | 0.02 | -0.47* | 0.14* | 0.11* | -0.02 | -0.04* | -0.07 | -0.05* | 1.00 |  |  |  |  |  |
| Mom smoked* | 0.25* | 0.07* | -0.08* | -0.20* | 0.11* | 0.01 | -0.39* | -0.30* | -0.33* | 0.13* | 0.07* | 0.28* | -0.04* | 0.10* | 1.00 |  |  |  |  |
| Dad smoked* | 0.31* | 0.05* | -0.12* | -0.15* | 0.10* | 0.00 | 0.03 | -0.20* | -0.24* | 0.15* | 0.11* | 0.33* | 0.05* | -0.06* | 0.61* | 1.00 |  |  |  |
| Birth weight | -0.16* | 0.03* | 0.08* | 0.07* | -0.04* | 0.12* | -0.25* | 0.08* | 0.08* | -0.05* | -0.03* | -0.08* | 0.09* | 0.10* | -0.13* | -0.12* | 1.00 |  |  |
| Breastfed | -0.24* | -0.03 | 0.19* | 0.13* | 0.02 | 0.05* | 0.28* | 0.26* | 0.24* | -0.05* | 0.00 | -0.12* | -0.15* | 0.15* | -0.32* | -0.22* | 0.04* | 1.00 |  |
| Gestational age | -0.05* | 0.01 | 0.03* | 0.06* | -0.01 | -0.03* | -0.09* | -0.01* | 0.05* | -0.02* | -0.04* | -0.07* | -0.04* | 0.05* | -0.03* | -0.04* | 0.57* | 0.03* | 1.00 |

Note. ^#^refers to Planned pregnancy, Mother smoked during pregnancy, Father smoked during pregnancy respectively. *indicates that the correlation between two variables is statistically significant. Correlation coefficient between continuous, categorical and binary variables was calculated using “polychoric” command, and statistical test was obtained using “pwcorr”, “tetrachoric”, “pbis”, “tab,chi2”, and “anova” command correspondingly in Stata. Month of birth was not significantly correlated with any other variables thus omitted in the above table..

| eTable 5. The association between the individual components of derived exposures (deprivation and threat) and psychopathology – using imputed data (n=10,709). | | | | |
| --- | --- | --- | --- | --- |
|  | Unadjusted model | | Adjusted model | |
|  | b | 95% CI | b | 95% CI |
| *Components of deprivation* |  |  |  |  |
| Family income | 0.18 | (-0.09, 0.46) | 0.16 | (-0.16, 0.47) |
| Area deprivation | 0.07 | (-0.31, 0.46) | 0.05 | (-0.34, 0.44) |
| Economic activity | 0.24 | (0.03, 0.45) | 0.17 | (-0.03, 0.38) |
| Housing tenure | 0.19 | (-0.06, 0.44) | 0.16 | (-0.13, 0.45) |
| Household social class |  |  |  |  |
| Neither parent in routine/manual occupation (reference) | - | - | - | - |
| Either parent in routine/manual occupation | -0.08 | (-0.79, 0.64) | -0.14 | (-0.81, 0.53) |
| Both parents in routine/manual occupation | 0.37 | (-0.37, 1.11) | 0.22 | (-0.53, 0.98) |
| Household academic qualification |  |  |  |  |
| NVQ 1 (reference) | - | - | - | - |
| NVQ 2 | 0.02 | (-1.04, 1.08) | 0.03 | (-0.94, 1.00) |
| NVQ 3 | 0.30 | (-0.93, 1.53) | 0.36 | (-0.76, 1.48) |
| NVQ 4 | -0.05 | (-1.12, 1.03) | 0.02 | (-1.00, 1.04) |
| NVQ 5 | 0.00 | (-2.05, 2.05) | 0.17 | (-1.73, 2.06) |
| Other | 0.49 | (-0.71, 1.70) | 0.33 | (-0.82, 1.48) |
|  |  |  |  |  |
| *Components of threat* |  |  |  |  |
| Interpartner violence | 0.23 | (-0.24, 0.69) | 0.11 | (-0.32, 0.53) |
| Smacking | -0.02 | (-0.36, 0.32) | 0.06 | (-0.25, 0.36) |
| Shouting | -0.04 | (-0.28, 0.21) | -0.02 | (-0.25, 0.21) |
| Physical restraint | 0.03 | (-1.40, 1.46) | 0.29 | (-1.09, 1.66) |

**References**

1. van Buuren S. Multiple imputation of discrete and continuous data by fully conditional specification. *Stat Methods Med Res*. 2007;16(3):219–42.

2. Collins LM, Schafer JL, Kam CM. A comparison of inclusive and restrictive strategies in modern missing data procedures. *Psychological Methods*. Dec 2001;6(4):330-351. doi:10.1037//1082-989x.6.4.330

3. Little R, Rubin DB. *Statistical analysis with missing data*. Wiley; 2002.

4. Austin PC, van Buuren S. The effect of high prevalence of missing data on estimation of the coefficients of a logistic regression model when using multiple imputation. *BMC Medical Research Methodology*. 2022/07/18 2022;22(1):196. doi:10.1186/s12874-022-01671-0

5. Sterne JA, White IR, Carlin JB, et al. Multiple imputation for missing data in epidemiological and clinical research: potential and pitfalls. *BMJ*. Jun 29 2009;338:b2393. doi:10.1136/bmj.b2393

6. Mostafa T, Narayanan M, Pongiglione B, et al. Missing at random assumption made more plausible: evidence from the 1958 British birth cohort. *Journal of Clinical Epidemiology*. 2021;doi:doi.org/10.1016/j.jclinepi.2021.02.019

7. White IR, Royston P, Wood AM. Multiple imputation using chained equations: issues and guidance for practice. *Statistics in Medicine*. 2011;30(4):377–99.

8. Moons KGM, Donders RART, Stijnen T, Harrell FEJ. Using the outcome for imputation of missing predictor values was preferred. *Journal of Clinical Epidemiology*. 2006;59(10):1092–101.
